# Combining quick sequential organ failure assessment score with heart rate variability may improve predictive ability for mortality in septic patients at the emergency department

**DOI:** 10.1371/journal.pone.0213445

**Published:** 2019-03-18

**Authors:** Sumanth Madhusudan Prabhakar, Takashi Tagami, Nan Liu, Mas’uud Ibnu Samsudin, Janson Cheng Ji Ng, Zhi Xiong Koh, Marcus Eng Hock Ong

**Affiliations:** 1 Yong Loo Lin School of Medicine, National University of Singapore, Singapore, Singapore; 2 Health Services and Systems Research, Duke-NUS Medical School, Singapore, Singapore; 3 Department of Emergency and Critical Care Medicine, Nippon Medical School Tama Nagayama Hospital, Tokyo, Japan; 4 Health Services Research Centre, Singapore Health Services, Singapore, Singapore; 5 Duke-NUS Medical School, Singapore, Singapore; 6 Department of Emergency Medicine, Singapore General Hospital, Singapore, Singapore; Hospital Universitari Bellvitge, SPAIN

## Abstract

**Background:**

Although the quick Sequential Organ Failure Assessment (qSOFA) score was recently introduced to identify patients with suspected infection/sepsis, it has limitations as a predictive tool for adverse outcomes. We hypothesized that combining qSOFA score with heart rate variability (HRV) variables improves predictive ability for mortality in septic patients at the emergency department (ED).

**Methods:**

This was a retrospective study using the electronic medical record of a tertiary care hospital in Singapore between September 2014 and February 2017. All patients aged 21 years or older who were suspected with infection/sepsis in the ED and received electrocardiography monitoring with ZOLL X Series Monitor (ZOLL Medical Corporation, Chelmsford, MA) were included. We fitted a logistic regression model to predict the 30-day mortality using one of the HRV variables selected from one of each three domains those previously reported as strong association with mortality (i.e. standard deviation of NN [SDNN], ratio of low frequency to high frequency power [LF/HF], detrended fluctuation analysis α-2 [DFA α-2]) in addition to the qSOFA score. The predictive accuracy was assessed with other scoring systems (i.e. qSOFA alone, National Early Warning Score, and Modified Early Warning Score) using the area under the receiver operating characteristic curve.

**Results:**

A total of 343 septic patients were included. Non-survivors were significantly older (survivors vs. non-survivors, 65.7 vs. 72.9, p <0.01) and had higher qSOFA (0.8 vs. 1.4, p <0.01) as compared to survivors. There were significant differences in HRV variables between survivors and non-survivors including SDNN (23.7s vs. 31.8s, p = 0.02), LF/HF (2.8 vs. 1.5, p = 0.02), DFA α-2 (1.0 vs. 0.7, P < 0.01). Our prediction model using DFA-α-2 had the highest c-statistic of 0.76 (95% CI, 0.70 to 0.82), followed by qSOFA of 0.68 (95% CI, 0.62 to 0.75), National Early Warning Score at 0.67 (95% CI, 0.61 to 0.74), and Modified Early Warning Score at 0.59 (95% CI, 0.53 to 0.67).

**Conclusions:**

Adding DFA-α-2 to the qSOFA score may improve the accuracy of predicting in-hospital mortality in septic patients who present to the ED. Further multicenter prospective studies are required to confirm our results.

## Introduction

Sepsis is a severe and life-threatening condition with high mortality and morbidity [1]. Several studies and guidelines suggest that early identification and immediate bundle management are essential components of sepsis management in order to improve sepsis patient’s outcome [2–4]. Thus, a quick, simple, non-invasive, and efficient risk stratification tool to identify high-risk patients may initiate the bundle management as recommended by the updated survival sepsis campaign bundle [3], especially in the early phase of sepsis during the emergency department (ED) setting.

The quick Sequential Organ Failure Assessment (qSOFA) score was recently introduced to identify patients with suspected infection using three physiological variables who are at greater risk for a poor outcome in non-intensive care unit settings [5]. The qSOFA score uses three criteria, assigning one point for low blood pressure (SBP≤100 mmHg), high respiratory rate (≥22 breaths per min), or altered mentation (Glasgow coma scale<15) [5]. Scoring of the physiological variables using qSOFA may have potential to predict adverse outcomes for septic patients and is widely used clinically worldwide [6–9]. Although these physiological variables alone may have high specificity in prediction of adverse outcomes for sepsis, a recent systematic review suggested that a positive qSOFA score had high specificity but low sensitivity in early detection of in-hospital mortality, [10]. Adding the variables that have strong association with mortality to qSOFA may improve current prediction models. Such a score could work as an early warning signal for impending septic deterioration in the ED population.

Several studies have reported the prognostic value of reduction of heart rate variability (HRV) in septic patients presenting to the ED [7, 11–15]. HRV is a noninvasive and quantitative test to evaluate autonomic function, which may be used as an early warning signal for impending patient deterioration in the ED population [16–18]. We therefore hypothesized that combining qSOFA with HRV variables improves predictive ability for mortality in septic patients at the ED.

The aim for the current study was to improve prediction models of 30-day in-hospital mortality for septic patients in the ED by combining HRV with the qSOFA score.

## Methods

This study was approved by the SingHealth Centralised Institutional Review Board (Ref: 2016/2858) with a waiver of informed patient consent.

### Design and setting

This was a retrospective analyses study using the electronic medical records of Singapore General Hospital (SGH), a tertiary care hospital in Singapore, between September 2014 and February 2017. In SGH, all patients were triaged by a trained expert nurse on arrival at the ED and were subsequently seen by an emergency physician. All patients who were aged 21 years or older and suspected with severe infection/sepsis in the ED and were able to receive electrocardiography monitoring with ZOLL X Series Monitor (ZOLL Medical Corporation, Chelmsford, MA) were included in the current study.

### Definitions and endpoint

Patient demographics and vital signs recorded in the patients’ electronic medical record were used for our analyses. Five minute one-lead electrocardiogram tracings were obtained from the X-Series Monitor. We loaded electrocardiogram tracings data into the HRV calculating software (Kubios version 2.2, Kuopio, Finland), and computed the time, frequency, and non-linear variables domain of the HRV[19]. We manually verified the QRS complexes of the electrocardiogram detected by the software. The R-R interval time series was then screened for rhythm, artifacts and ectopic beats. If artifacts or ectopic beats were few (<5), they were removed from the R-R interval time series. We excluded the patients with non-sinus rhythm and/or ectopic beats from the current study.

The time domain of the HRV variables are statistical calculations of consecutive R-R time intervals (NN intervals), such as mean NN (mean NN), standard deviation of NN (SD NN), standard deviation of heart rate (SD HR), root mean square of the differences between adjacent NN intervals (RMSSD), the baseline width of the minimum square difference triangular interpolation of the highest peak of the histogram of all NN intervals (TINN). A recent study suggested that SDNN has the strongest relationship with mortality among septic patients [20]. Frequency domain HRV variables are based on spectral analysis: very low frequency (VLF), low frequency (LF), high frequency (HF), ratio of LF to HF power (LF/HF). Several studies suggested that LF/HF has potential to predict short-term deterioration in emergency department patients with sepsis [21–23]. The non-linear domain includes detrended fluctuation analysis (DFA) α-1 and α-2, standard deviations of Poincare plot, and sample entropy. Several studies indicated that regulators of the cardiovascular system interact in a non-linear way [24, 25], and DFA α-2 has strong association with mortality in septic patients [7].

For comparison with qSOFA, we also estimated the following two scoring systems from the ED and electronic medical record: the Modified Early Warning Score (MEWS)[26] and the National Early Warning Score (NEWS) [27]. The two Early Warning Scoring systems consist of 5 (MEWS) or 6 (NEWS) physiological variables; respiratory rate, temperature, systolic blood pressure, heart rate, and mental status (and oxygen saturations for NEWS) [26, 27].

The primary endpoint for the current study was all-cause 30-day mortality.

### Statistical analysis

Categorical variables of the patients’ baseline characteristics were presented with percentage, and compared using a Chi-square or Fisher’s test. Continuous variables were presented with mean and standard deviation (SD), and compared using student’s t test. We fitted a logistic regression model to predict the 30-day mortality using from one of the three domains of the HRV variables (i.e. SDNN [20], LF/HF [21–23], DFA α-2 [7]) in addition to the qSOFA score [28]. The predictive accuracy was assessed using the area under the receiver operating characteristic curve (AUC) and presented with c-statistic with 95% confidential interval (CI). The statistical significance threshold was a P value of less than 0.05. All analyses were carried out using SPSS software (IBM Corp., Armork, NY, USA, version 23).

## Results

During the study period, 343 patients met the inclusion criteria for this study. Table 1 shows the characteristics of the patients in the current study. There was no significant difference in the proportion of gender, race, source of infection, medical and drug history between survivors and non- survivors. However, non-survivors were significantly older and had higher qSOFA, NEWS, and MEWS scores as compared to survivors.

**Table 1:** Background characteristics of the patients.

Variable	Survivors (n = 257)	Non-survivors (n = 86)	*P*-value
Age, mean (SD)	65.7 (15.8)	72.9 (15.0)	<0.01
Gender, male	137 (53.3)	37 (43.0)	0.11
Race			0.97
Chinese	190 (73.9)	63 (73.3)	
Indian	35 (13.6)	13 (15.1)	
Malay	21 (8.2)	6 (7.0)	
Other	11 (4.3)	4 (4.7)	
Source of infection			0.12
Respiratory	74 (28.8)	33 (38.4)	
Urinary Tract	60 (23.3)	15 (17.4)	
abdominal	41 (16.0)	6 (7.0)	
Musculoskeletal	10 (3.9)	4 (4.7)	
Others/Unknown	72 (28.0)	28 (32.6)	
Medical history			
Ischemic heart disease	69 (26.8)	26 (30.2)	0.58
Diabetes	105 (40.9)	31 (36.0)	0.45
Hypertension	148 (57.6)	46 (53.5)	0.53
Cancer	67 (26.2)	32 (37.2)	0.06
Previous sepsis admission	107 (41.6)	36 (41.9)	1.00
Drug history			
Beta-blocker	91 (35.4)	25 (29.1)	0.30
Digoxin	10 (3.9)	3 (3.5)	1.00
Calcium channel blocker	69 (26.8)	21 (24.4)	0.78
Amiodarone	3 (1.2)	1 (1.2)	1.00
qSOFA, mean (SD)	0.8 (0.7)	1.4 (0.9)	<0.01
NEWS, mean (SD)	6.1 (2.8)	8.0 (3.3)	<0.01
MEWS, mean (SD)	4.7 (1.9)	5.3 (2.0)	0.01

MEWS, Modified Early Warning Score; NEWS, National Early Warning Score; qSOFA, Quick Sequential Organ Failure Assessment; SD, standard deviation

As shown in Table 2, lower systemic blood pressure, higher respiratory rate, and worse consciousness levels were observed in non-survivors as compared to survivors. There were significant differences in HRV variables between survivors and non-survivors including SDNN (survivors vs. non-survivors, 23.7s vs. 31.8s, p = 0.02), LF/HF (2.8 vs. 1.5, p = 0.02), DFA α-2 (1.0 vs. 0.7, P < 0.01).

**Table 2:** Initial vital signs and heart rate variabilities at emergency department between survivors and non-survivors.

Variable	Survivors (n = 257)	Non-survivors (n = 86)	*P*-value
Heart rate (beats/min)	113.7 (24.2)	112.8 (24.3)	0.76
Respiratory rate (breaths/min)	20.3 (7.8)	22.4 (5.3)	0.02
Systolic BP (mmHg)	114.9 (33.8)	102.6 (30.6)	<0.01
Diastolic BP (mmHg)	63.4 (19.7)	59.3 (17.2)	0.09
GCS score	13.5 (2.9)	11.9 (4.0)	<0.01
Temperature (°C)	37.9 (2.5)	37.4 (1.3)	0.09
**HRV measures**			
**Time domain**			
Mean RR (s)	579.0 (132.8)	583.3 (153.2)	0.80
SD RR (s)	23.7 (26.4)	31.8 (33.3)	0.02
Mean HR (bpm)	108.9 (22.4)	109.3 (24.0)	0.88
SD HR (bpm)	4.8 (5.9)	6.6 (6.8)	0.02
RMSSD (s)	27.3 (39.0)	43.2 (50.3)	<0.01
NN50 (count)	48.7 (115.8)	64.5 (112.3)	0.27
pNN50 (%)	7.7 (18.1)	10.7 (18.7)	0.19
RR triangular index	4.2 (3.8)	4.3 (4.8)	0.91
TINN	146.1 (155.2)	200.3 (173.7)	0.01
Total power (ms2)	691.5 (2124.1)	1037.4 (2920.6)	0.24
**Frequency domain**			
VLF power (ms2)	170.3 (515.7)	215.9 (756.3)	0.53
LF power (ms2)	166.6 (569.8)	240.3 (751.4)	0.34
HF power (ms2)	352.2 (1184.7)	577.2 (1555.5)	0.16
LF power norm (n.u.)	48.5 (28.6)	32.9 (26.3)	<0.01
HF power norm (n.u.)	50.8 (28.1)	66.2 (26.0)	<0.01
LF/HF	2.8 (4.8)	1.5 (4.4)	0.02
**Non-linear domain**			
Poincare plot SD1 (ms)	19.3 (27.6)	30.6 (35.6)	<0.01
Poincare plot SD2 (ms)	25.6 (26.7)	31.4 (32.2)	0.10
Approximate entropy	1.0 (0.3)	1.0 (0.3)	0.51
Sample entropy	1.1 (0.5)	1.0 (0.5)	0.22
DFA, α-1	0.7 (0.4)	0.5 (0.3)	<0.01
DFA, α-2	1.0 (0.4)	0.7 (0.4)	<0.01

Data were presented with mean (standard deviation).

BP, blood pressure; GCS, Glasgow Coma Scale; mean RR, average width of the RR interval; SD RR, standard deviation of all RR intervals; HR, heart rate; RMSSD, root mean square of differences between adjacent RR intervals; NN50, number of consecutive RR intervals differing by more than 50 ms; pNN50, percentage of consecutive RR intervals differing by more than 50 ms; TINN, baseline width of a triangle fit into the RR interval histogram using a least squares; VLF, very low frequency; LF, low frequency; HF, high frequency; norm, normalized; LF/HF, ratio of LF power to HF power; DFA, detrended fluctuation analysis.

Table 3 shows the c-statistic of each of the predicting model and scores for the primary outcome of all-cause 30-day mortality.

**Table 3:** Comparison of the models to predict all-cause 30-day mortality.

Prediction models	AUC	Standard. Error	95% Confidence Interval	
			Lower Bound	Upper Bound
MEWS	0.60	0.04	0.53	0.67
NEWS	0.67	0.03	0.61	0.74
qSOFA	0.68	0.03	0.62	0.75
qSOFA + SD RR	0.71	0.03	0.64	0.77
qSOFA +LF/HF	0.74	0.03	0.68	0.80
qSOFA + DFA, α-2	0.76	0.03	0.70	0.82
qSOFA+ SDNN +LF/HF + DFA, α-2	0.76	0.03	0.70	0.82

AUC, Area under the curve; DFA, detrended fluctuation analysis, LF/HF, ratio of LF power to HF power; qSOFA, Quick Sequential Organ Failure Assessment; SD RR, standard deviation of all RR intervals.

**Fig 1:**
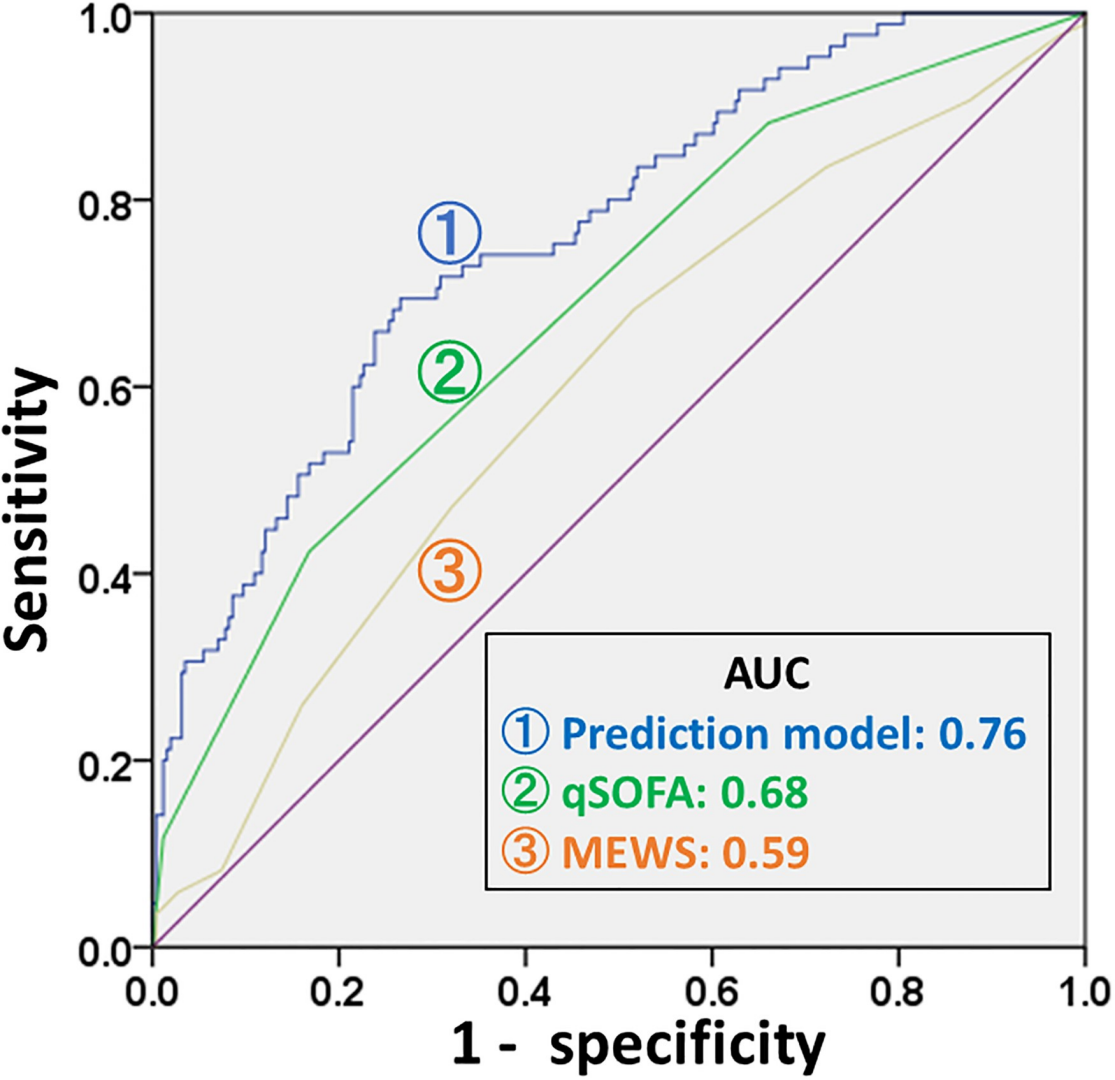
Area under the receiver operating characteristic curves to predict all-cause 30-day mortality of our predicting model and other scoring systems. MEWS, Modified Early Warning Score; qSOFA, Quick Sequential Organ Failure Assessment.

The model of qSOFA with DFA, α-2 represented AUC of 0.76, which was better than that of MEWS at 0.59 (95% CI, 0.53 to 0.67) (Fig 1). The c-statistic of the qSOFA with DFA α-2 did not improve significantly even if we include the other two HRV variables (i.e. SDNN and LF/HF) into the model (i.e. qSOFA + DFA α-2, AUC 0.76, 95% CI 0.70–0.82 vs. qSOFA+ SDNN +LF/HF + DFA α-2, AUC 0.76, 95% CI 0.70–0.82).

## Discussions

The results of the current study suggested that the model created from HRV variable (especially DFA α-2) in addition to qSOFA score may improve the accuracy in predicting all-cause 30-day mortality in patients who present to the ED with suspicion of infection/sepsis. This prediction model may work as simple, non-invasive, and efficient risk stratification tool to identify high-risk septic patients at the ED.

Scoring of the physiological variables, such as using MEWS, NEWS, and qSOFA, may have potential to predict adverse outcomes for septic patients. The two Early Warning Scoring systems consist of 5 or 6 physiological variables which may predict the deterioration of patient’s clinical course. The qSOFA score was recently introduced to identify patients with suspected infection using three physiological variables who are at greater risk for a poor outcome in non-intensive care unit settings [5]. It is still a matter of controversy which scoring system is better [29–31]. Although these scoring systems alone may have potential for acceptable prognostic accuracy among homogeneous populations of a certain disease, recent systematic review and meta-analysis studies suggest that the neither the Early Warning Scores nor qSOFA accurately predict mortality in patients with suspected infection/sepsis [32, 33]. Although these physiological variables alone may have high specificity in prediction of adverse outcomes for sepsis, low sensitivity may limit the utility of these scores [10]. However, variables of ED prediction models should be simple, easy and non-invasive to obtain, in the time-limited clinical setting of the ED. Our results showed that the model of qSOFA with DFA α-2 represented better predictive ability than that of MEWS for all-cause 30-day mortality. More importantly, our model does not require additional, invasive, time-consuming, nor unvalidated variables to estimate.

The rationale for and feasibility of evaluating continuous HRV monitoring in the ED has been well described in previous studies[7, 22, 34]. Several studies had suggested that HRV changes may present as the earliest measurements before apparent clinical symptoms emerge [23, 35, 36]. There is close interaction between the parasympathetic nervous system (which can be detected by HRV) and the immune system [37–41]. The HRV variables change with the release of cytokines and other inflammatory mediators, such as soluble tumor necrosis factor-α receptors [37, 38], interleukin-6 [38, 41, 42], and C-reactive protein [39, 40, 42]. In our study, there were significant differences in most of the HRV variables between survivors and non-survivors including the variables we included in our predictive model (i.e. SDNN, LF/HF, DFA α-2). Without requirement of further blood samples, HRV variables can be feasibly obtained non-invasively at the bedside from ED patients.

Several limitations must be considered in the interpretation of the current results. First, we could not include all sepsis patient who visited our ED in the current study. The recruitment of the patients were sometimes limited due to office hours and depending on available manpower. Moreover, there is no reference standard to determine the septic patient, and we had enrolled patients who had clinically suspected sepsis/infection based on clinical diagnosis. Thus, we might have excluded patients who were septic or included conditions other than sepsis in the current study. Second, we determined HRV variables from one of each of three domains (i.e. SDNN, LF/HF, DFA α-2) to implement in our predictive model based on the results of previous studies. We choose this approach to prevent overfitting of the model and allow reproducibility of the study. Although we selected our three variables based on our literature review, there might have other HRV variables suitable for a multivariate regression model. Finally, the study was conducted retrospectively in an observational manner without randomization that focused only on septic patients. Therefore, the results might not be generalized to other settings and a cause-effect relationship cannot be established. External validation studies are required in larger multicenter prospective studies to confirm our results.

## Conclusions

Adding HRV variables, especially DFA α-2, to the qSOFA score may improve the accuracy of predicting in-hospital mortality in septic patients who present to the ED. Further multicenter prospective studies are required to confirm our results.
